# UniProt: the universal protein knowledgebase

**DOI:** 10.1093/nar/gkw1099

**Published:** 2016-11-28

**Authors:** The UniProt Consortium, Alex Bateman, Maria Jesus Martin, Claire O’Donovan, Michele Magrane, Emanuele Alpi, Ricardo Antunes, Benoit Bely, Mark Bingley, Carlos Bonilla, Ramona Britto, Borisas Bursteinas, Hema Bye-A-Jee, Andrew Cowley, Alan Da Silva, Maurizio De Giorgi, Tunca Dogan, Francesco Fazzini, Leyla Garcia Castro, Luis Figueira, Penelope Garmiri, George Georghiou, Daniel Gonzalez, Emma Hatton-Ellis, Weizhong Li, Wudong Liu, Rodrigo Lopez, Jie Luo, Yvonne Lussi, Alistair MacDougall, Andrew Nightingale, Barbara Palka, Klemens Pichler, Diego Poggioli, Sangya Pundir, Luis Pureza, Guoying Qi, Alexandre Renaux, Steven Rosanoff, Rabie Saidi, Tony Sawford, Aleksandra Shypitsyna, Elena Speretta, Edward Turner, Nidhi Tyagi, Vladimir Volynkin, Tony Wardell, Kate Warner, Xavier Watkins, Rossana Zaru, Hermann Zellner, Ioannis Xenarios, Lydie Bougueleret, Alan Bridge, Sylvain Poux, Nicole Redaschi, Lucila Aimo, Ghislaine Argoud-Puy, Andrea Auchincloss, Kristian Axelsen, Parit Bansal, Delphine Baratin, Marie-Claude Blatter, Brigitte Boeckmann, Jerven Bolleman, Emmanuel Boutet, Lionel Breuza, Cristina Casal-Casas, Edouard de Castro, Elisabeth Coudert, Beatrice Cuche, Mikael Doche, Dolnide Dornevil, Severine Duvaud, Anne Estreicher, Livia Famiglietti, Marc Feuermann, Elisabeth Gasteiger, Sebastien Gehant, Vivienne Gerritsen, Arnaud Gos, Nadine Gruaz-Gumowski, Ursula Hinz, Chantal Hulo, Florence Jungo, Guillaume Keller, Vicente Lara, Philippe Lemercier, Damien Lieberherr, Thierry Lombardot, Xavier Martin, Patrick Masson, Anne Morgat, Teresa Neto, Nevila Nouspikel, Salvo Paesano, Ivo Pedruzzi, Sandrine Pilbout, Monica Pozzato, Manuela Pruess, Catherine Rivoire, Bernd Roechert, Michel Schneider, Christian Sigrist, Karin Sonesson, Sylvie Staehli, Andre Stutz, Shyamala Sundaram, Michael Tognolli, Laure Verbregue, Anne-Lise Veuthey, Cathy H Wu, Cecilia N Arighi, Leslie Arminski, Chuming Chen, Yongxing Chen, John S Garavelli, Hongzhan Huang, Kati Laiho, Peter McGarvey, Darren A Natale, Karen Ross, C R Vinayaka, Qinghua Wang, Yuqi Wang, Lai-Su Yeh, Jian Zhang

**Affiliations:** 1 European Molecular Biology Laboratory, European Bioinformatics Institute (EMBL-EBI), Wellcome Genome Campus, Hinxton, Cambridge CB10 1SD, UK; 2 SIB Swiss Institute of Bioinformatics, Centre Medical Universitaire, 1 rue Michel Servet, 1211 Geneva 4, Switzerland; 3 Protein Information Resource, Georgetown University Medical Center, 3300 Whitehaven Street NW, Suite 1200, WA 20007, USA; 4 Protein Information Resource, University of Delaware, 15 Innovation Way, Suite 205, Newark DE 19711, USA

## Abstract

The UniProt knowledgebase is a large resource of protein sequences and associated detailed annotation. The database contains over 60 million sequences, of which over half a million sequences have been curated by experts who critically review experimental and predicted data for each protein. The remainder are automatically annotated based on rule systems that rely on the expert curated knowledge. Since our last update in 2014, we have more than doubled the number of reference proteomes to 5631, giving a greater coverage of taxonomic diversity. We implemented a pipeline to remove redundant highly similar proteomes that were causing excessive redundancy in UniProt. The initial run of this pipeline reduced the number of sequences in UniProt by 47 million. For our users interested in the accessory proteomes, we have made available sets of pan proteome sequences that cover the diversity of sequences for each species that is found in its strains and sub-strains. To help interpretation of genomic variants, we provide tracks of detailed protein information for the major genome browsers. We provide a SPARQL endpoint that allows complex queries of the more than 22 billion triples of data in UniProt (http://sparql.uniprot.org/). UniProt resources can be accessed via the website at http://www.uniprot.org/.

## INTRODUCTION

Protein science is entering a new era that promises to unlock many of the mysteries of the cell's inner workings. Next generation sequencing is transforming the way that we access DNA information and, as the variety of protein assays that can be linked to a DNA or RNA read-out grows, we are gaining protein information at an increasing rate. For example, ribosomal profiling experiments are telling us when and where ribosomes bind for translation while CHIP-seq and CRAC give us information on the DNA and RNA binding properties of proteins. We are also gaining new insights into the mechanics of large assemblies of proteins through the incredible strides being made in electron microscopy technology. However, this wealth of molecular data will be worth little without it being available to and interpretable by the scientific community.

UniProt is a long-standing collection of databases that enable scientists to navigate the vast amount of sequence and functional information available for proteins. The UniProt Knowledgebase (UniProtKB) is the central resource that combines UniProtKB/Swiss-Prot and UniProtKB/TrEMBL. UniProtKB/Swiss-Prot contains over 550 000 sequences that have been created by our expert biocuration team. For these entries experimental information has been extracted from the literature and organized and summarized, greatly easing scientists access to protein information. UniProtKB/TrEMBL provides a further 60 million sequences that have been largely derived from high throughput sequencing of DNA. These entries are annotated by our rule based automatic annotation systems. We also provide a series of UniRef databases that provide sequence sets trimmed at various levels of sequence identity (1,2). Finally we provide the UniProt Archive (UniParc) that provides a complete set of known sequences, including historical obsolete sequences (3).

In this article we describe the major developments that we have made since our last update published in this journal in 2014 (4). These updates have helped to improve the scalability and usability of UniProt for our end users. We also include a call to all life science authors to include UniProt accession numbers for proteins within their papers to help improve the connections between the literature and scientific databases.

## PROGRESS AND NEW DEVELOPMENTS

### Identifying redundant proteomes

**Figure 1.**
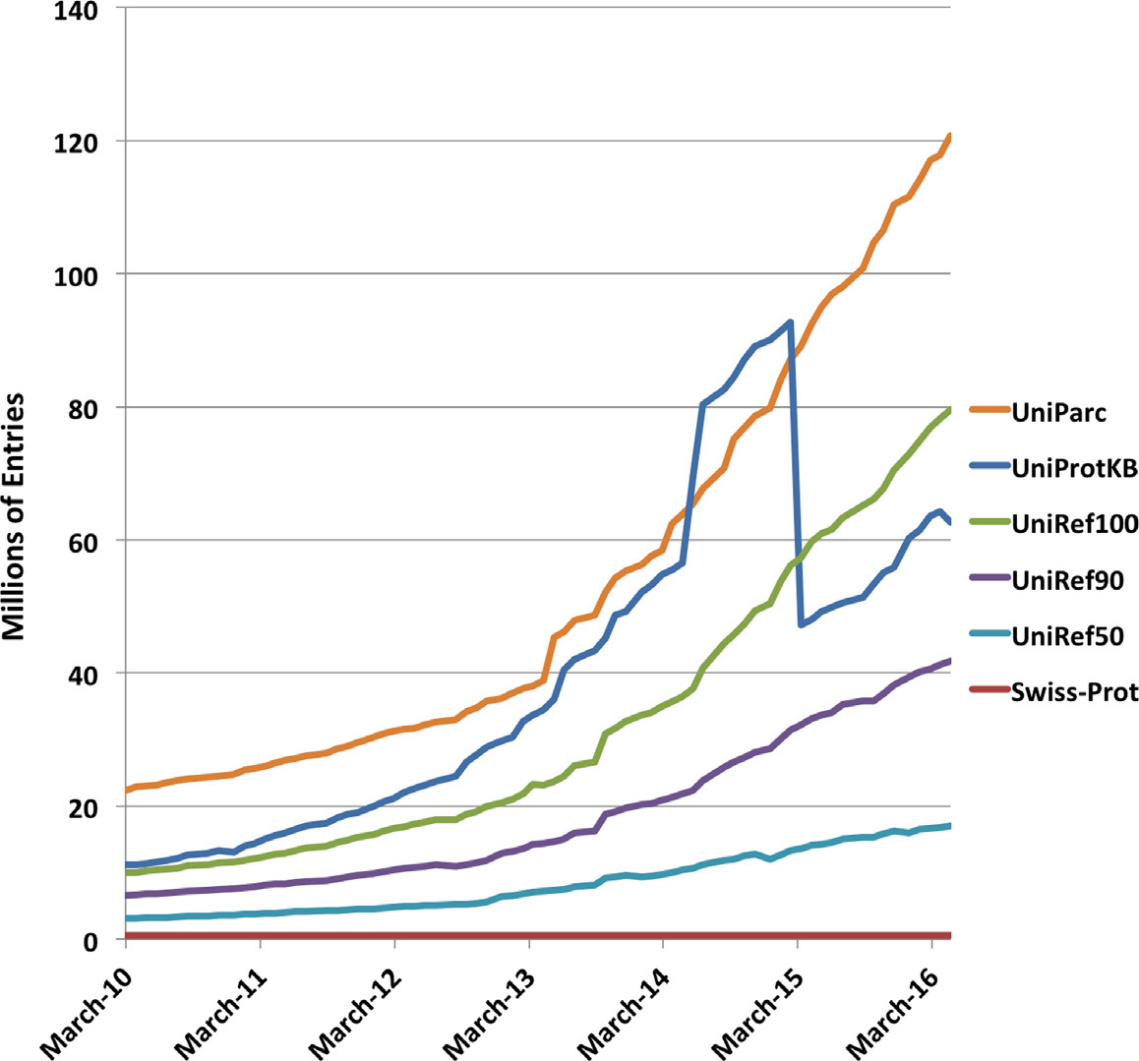
Growth of the number of sequences in UniProt databases. The blue line shows the growth in UniProtKB/TrEMBL entries from January 2010 to date. The sharp drop in UniProtKB entries corresponds to the proteome redundancy minimization (PRM) procedure implemented in March 2015. Note that the post-PRM growth in UniProtKB is no longer exponential.

The accelerating growth of sequenced genomes poses great challenges for databases with their major growth being driven by sequencing of very similar and almost identical strains of the same bacterial species. UniProtKB saw an exponential growth in the 2014–2015 time period, with April 2014 seeing a very high increase, reaching a peak of 90 million sequences with a high level of redundancy (e.g. 4080 proteomes of *Staphylococcus aureus* comprising 10.88 million proteins), see Figure 1. While this wealth of protein information presents our users with new opportunities for proteome-wide analysis and interpretation, it also creates challenges in capturing, searching, preserving and presenting proteome data to the scientific community. In 2015, we introduced a method to identify and remove highly redundant bacterial proteomes within species groups, for example, strains of *Escherichia coli*, in UniProtKB while still making them available for users in UniParc. Briefly, the method identifies redundant proteomes by performing pairwise alignments of sets of sequences for pairs of proteomes and subsequently applies graph theory to find dominating sets that result in removal of proteomes that cause a minimal loss of information. Approximately 47 million proteins were removed from UniProtKB in March 2015. If this procedure were not being applied UniProtKB would now contain over 217 million sequences compared to the current 65 million. We have recently extended this approach to fungal species where there has been a similar if not as large (as yet) explosion in sequencing of highly similar genomes. As a result of this, ∼1 million fungal UniProtKB entries were deprecated in release 2016_08.

### Growth of reference proteomes

The UniProt proteome pages (http://www.uniprot.org/proteomes/) provide access to proteomes for over 56 000 (56 317, release 2016_07) species with completely sequenced genomes. The majority of these proteomes are based on the translation of genome sequence submissions to the International Nucleotide Sequence Database Consortium (INSDC) but also include predictions from Ensembl, RefSeq reference genomes and vector/parasite-specific databases like VectorBase (5) and WormBase ParaSite (6). Some proteomes may also include protein sequences based on high quality cDNAs that cannot be mapped to the current genome assemblies (due to sequencing errors or gaps) and have been manually reviewed following supporting evidence, and/or careful analysis of homologous sequences from closely related organisms. Proteomes can include both manually reviewed (UniProtKB/Swiss-Prot) and unreviewed (UniProtKB/TrEMBL) entries. The proportion of reviewed entries varies between proteomes, and is obviously greater for the proteomes of intensively curated model organisms. For example, the proteomes for *Saccharomyces cerevisiae 288C* and *E. coli strain K12* consist entirely of reviewed entries. The unreviewed records are updated by our automatic annotation systems for every release, ensuring consistency and up-to-date annotations. To help in tracking and displaying proteomes, we use a proteome identifier that uniquely identifies the set of proteins corresponding to a single assembly of a completely sequenced genome. An example of a proteome can be found here: http://www.uniprot.org/proteomes/UP000005640.

It is important to realize that there may be additional records for a particular species which do not belong to the defined proteome. For more information, please read http://www.uniprot.org/help/proteome.

**Figure 2.**
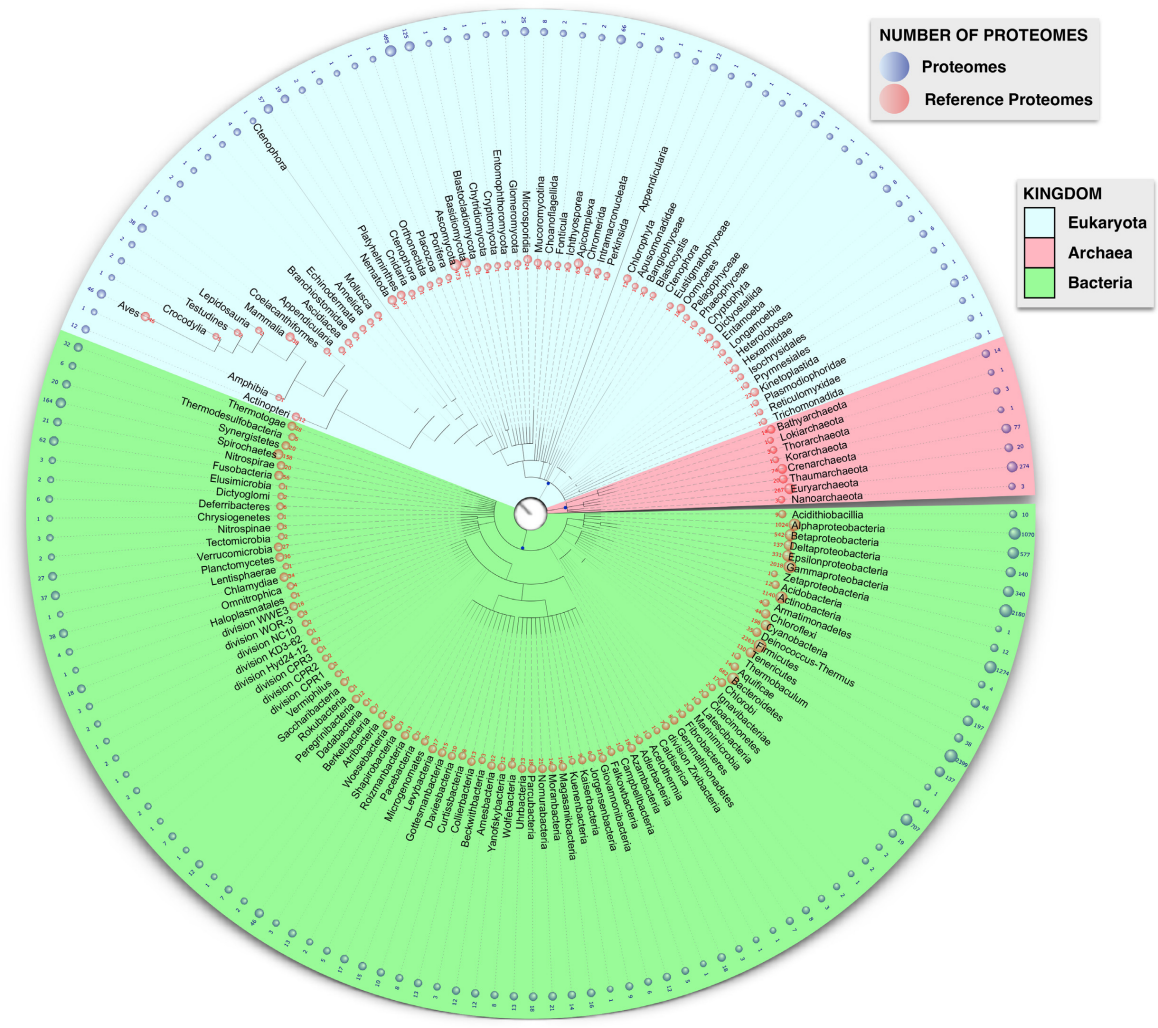
The distribution of proteomes and reference proteomes across the tree of life.

As the number of new proteomes increases, it is important to organize the data in a way that allows users to effectively navigate the proteomes while keeping relevant and pertinent information easily accessible. To achieve this, UniProt provides reference proteomes to give a selection of species representing a broad coverage of the tree of life. These reference proteomes are selected via consultation with the research community or computationally determined from proteome clusters (7) using an algorithm that considers the best overall annotation score, an automatically calculated score which provides a heuristic measure of the annotation content of a proteome. There are currently 5,631 reference proteomes representing a cross-section of the taxonomic diversity found in UniProtKB (Figure 2). They are the focus of both manual and automatic annotation, aiming to provide the best annotated protein sets for the selected species. They include model organisms and other proteomes of interest to biomedical and biotechnological research. We very much welcome feedback from our user community on our current list of reference proteomes and suggestions for new candidates.

### Pan proteomes

To complement the growing set of reference proteomes, UniProt has developed pan proteomes analogous to the pan genome concept (8). A pan proteome is the full set of proteins expressed by a group of highly related organisms (e.g. multiple strains of the same bacterial species). Pan proteomes provide a representative set of all the sequences within a taxonomic group and capture unique sequences not found in the group's reference proteome. UniProtKB pan proteomes encompass all non-redundant proteomes and are aimed at users interested in phylogenetic comparisons and the study of genome evolution and gene diversity.

For each reference proteome cluster, also known as representative proteome group (7), a pan proteome is the set of all the sequences in the reference proteome plus the unique protein sequences that are found in other species or strains of the cluster but not in the reference proteome. Details on how we compute pan proteomes can be found here: http://pir.georgetown.edu/rps/pp.shtml.

Pan proteomes are available as files of FASTA formatted sequences on the FTP site. On the UniProt website's proteomes pages, you will see a link to download the pan proteome file when a proteome has proteins that are part of a larger pan proteome.

### Manual curation progress

Expert literature-based curation is stored in the UniProtKB/Swiss-Prot section of the UniProt Knowledgebase (UniProtKB) and constitutes a cornerstone of UniProt. UniProtKB/Swiss-Prot provides high quality annotation for experimentally characterized proteins and represents the world's most comprehensive catalogue of information on proteins. It is composed of more than 550 000 curated proteins, including all protein-coding genes for a number of key organisms such as human, *S. cerevisiae, Schizosaccharomyces pombe, Bacillus subtilis* and *E. coli* and contains in-depth information extracted from more than 210 000 publications that have been fully curated. In the last year we have added 3183 new entries to UniProtKB/Swiss-Prot (releases 2015_10 to 2016_09), which represents the growth in newly characterized proteins.

In spite of the rise of big data and high-throughput technologies in recent years, which have shifted a number of paradigms in the scientific community, expert curation is by far the most reliable method to report gold-standard information and provide an up-to-date knowledgebase containing experimental information. With more than a million articles indexed every year in PubMed, strict prioritization of articles and proteins for curation is crucial. A key challenge is the identification of relevant high-quality articles that allow for the comprehensive curation of a protein and literature selection is made according to well-established criteria which have been documented elsewhere (see (9) for a description of categories of literature that are prioritized).

Literature curation of post-translational modifications (PTMs) and their consequences is a priority due to their crucial role in generating protein complexity and regulating protein activity, thus controlling many biological processes. Curation of experimentally determined PTMs from the literature is important as many PTMs cannot be reliably predicted by computational tools. Over the years, experimental PTMs have been catalogued in UniProtKB/Swiss-Prot, a catalog that can be used both as a high-quality training set for development and enhancement of bioinformatics algorithms, and as an essential library for the identification of proteins by proteomics. Priority is given to articles that describe new PTMs and/or characterize the effects of modifications. In addition, to complement this approach, we have developed a semi-automatic pipeline for integration of high-throughput proteomics data that is distinct from expert curation and which adds PTMs from manually evaluated large-scale proteomics publications (10).

The tubulin-alpha and -beta chains, which regulate microtubule properties and functions, provide a good example of the breadth of PTM curation in UniProtKB. A number of PTMs have been identified on tubulin chains, such as acetylation, phosphorylation, tyrosination/detyrosination, nitration, polyglutamylation and polyglycylation. Interestingly, while these modifications have been known for many years, the roles they play are only now starting to be understood. Recent advances in the field suggest the existence of a tubulin code, similar to the histone code, with a combination of PTMs that are interpreted by reader proteins (11).

**Figure 3.**
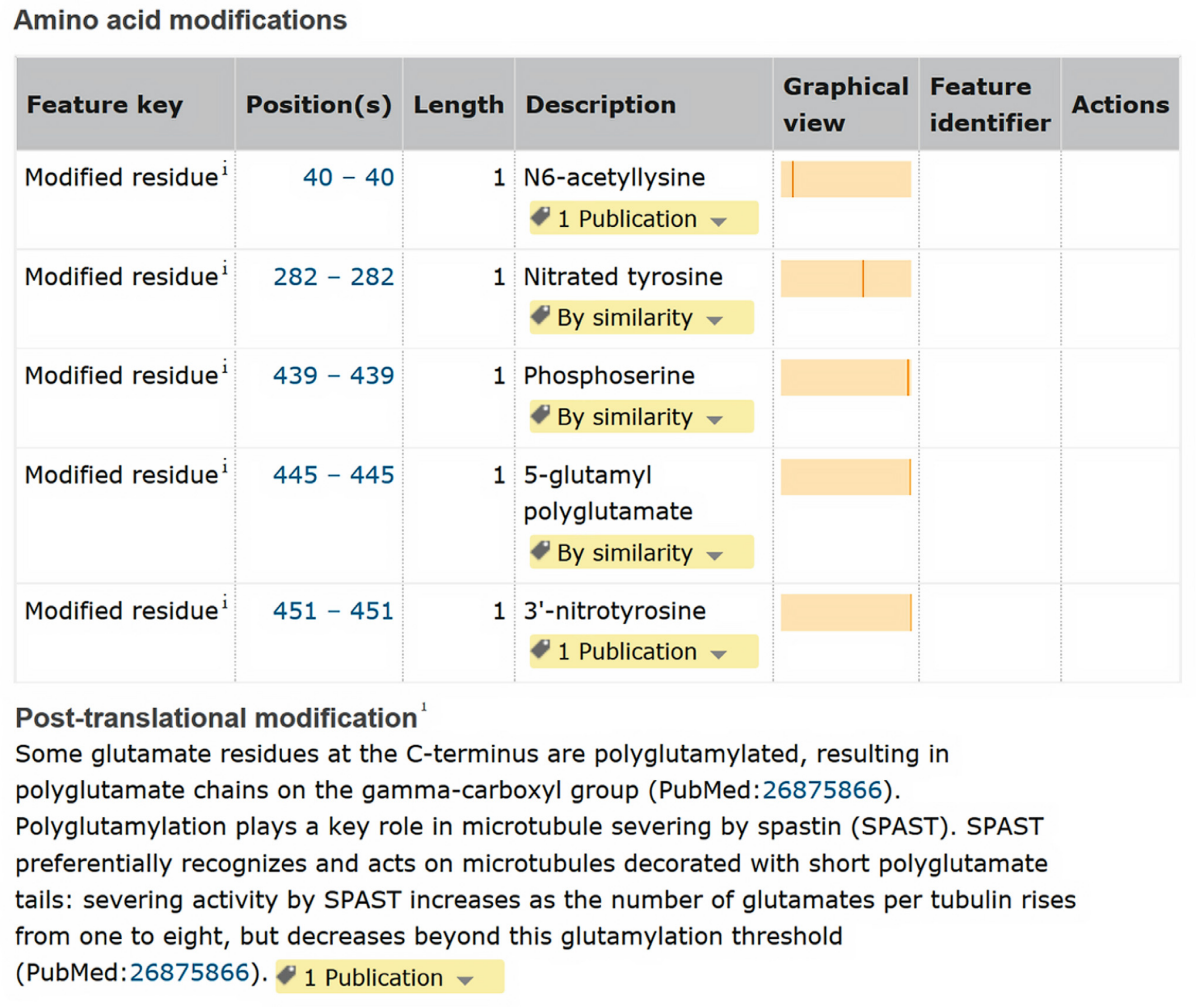
Screenshot of a part of the ‘PTM/Processing’ section of human TUBA1A entry (UniProtKB Q71U36, http://www.uniprot.org/uniprot/Q71U36)

In UniProtKB entries, PTM information is stored in the ‘PTM/Processing’ section, which describes the modified residues and a summary of what is known about the PTMs. Figure 3 shows the ‘PTM/Processing’ section for the human tubulin alpha-1A isotype (UniProtKB Q71U36). Descriptions of the modified residues are structured in a machine-readable format with the use of standardized vocabularies being essential to organize knowledge for subsequent retrieval. Four annotation types describe different types of amino acid modifications: modified residue, Disulfide bond, Cross-link and Glycosylation. Each PTM annotation is associated with a controlled vocabulary established in collaboration with the RESID database (12) and PSI-MOD (13). A list of all terms used in UniProtKB is available in the ptmlist.txt document (http://www.uniprot.org/docs/ptmlist).

Traceability of data is fundamental. Every new piece of knowledge is associated with the original source of the information and the type of supporting evidence, using the Evidence and Conclusion Ontology (14). In the example shown in Figure 3, detyrosination, acetylation and nitration are supported by experimental evidence and the source references are displayed. In contrast, polyglutamylation (5-glutamyl polyglutamate) is inferred by similarity to a related protein where the modification has been experimentally determined, and the source entry (UniProtKB P68369) is indicated. Interestingly, while polyglutamylation, which consists of the formation of glutamate side chains on specific glutamate residues in the C-terminal tail of both tubulin-alpha and -beta chains, is well-known, the precise positions are still unclear for most proteins. For tubulin-alpha entries, only one site has been unambiguously identified in mouse.

In addition to describing amino acid modifications on the protein sequence in a structured format, the ‘PTM’ subsection provides a comprehensive summary of available PTM information (Figure 3). We describe, for example, that tubulin polyglutamylation acts as a rheostat for microtubule severing by spastin (SPAST, UniProtKB Q9UBP0). Spastin specifically recognizes and cuts microtubules that are polyglutamylated: severing activity by spastin increases as the number of glutamates per tubulin rises from one to eight and decreases beyond this glutamylation threshold (15). We also describe the glycylation modification and explain why human tubulin is only monoglycylated and not polyglycylated as in other organisms, due to the absence of a functional TTLL10 enzyme in human (UniProtKB Q6ZVT0).

We attach specific importance to accuracy of knowledge. Information is reported in appropriate entries, which can be challenging in the case of tubulins where researchers often work with extracts containing different isotypes, making the task of precisely assigning information to the appropriate protein-coding gene difficult. When experiments are made on specific tubulin isotypes, information is reported in the entries corresponding to the relevant isotypes. For example, Edde *et al.* determined the protein sequence of a peptide that is polyglutamylated in mouse (16). The peptide sequence corresponded to Tuba1a and Tuba1b proteins and this information is reported in both of these entries (UniProtKB P68369 and P05213). In cases where experiments are done with tubulin extracts containing a mixture of different tubulin isotypes, information is reported in all of the tubulin isotypes that contain the modification: for example, Barisic *et al.* (17) performed experiments in human cells and information about detyrosination is reportedin all human tubulin-alpha isotypes that contain a tyrosine residue at the C-terminus (UniProtKB Q71U36, P68363, Q9BQE3, Q13748 and Q6PEY2).

Annotation consistency and completeness is essential. In the course of PTM curation, curators also check that the annotation content of enzymes that mediate modifications is up-to-date. For example, detailed information can be found on proteins that mediate tubulin polyglutamylation (UniProtKB O95922, Q9BWV7, Q14679, Q6EMB2, Q8N841, Q6ZT98, Q3SXZ7, Q8NHH1, A6NNM8) and acetylation (UniProtKB Q5SQI0). We also describe proteins that ‘read’ modifications, such as the tubulin-severing ATPase spastin (UniProtKB accession Q9UBP0), which specifically acts on microtubules that are polyglutamylated.

The constant effort over the last 30 years has given rise to a unique resource in terms of PTM information. In total, more than 450 different types of PTMs are described in UniProtKB/Swiss-Prot, 45 000 experimentally proven modification sites are curated and more than 22 000 UniProtKB/Swiss-Prot entries contain experimental information on PTMs.

### Including UniProt accession numbers in scientific papers

The biomedical literature is vast, with over one million papers being added to PubMed every year. As mentioned above, UniProt curators triage these and select relevant papers to create and update our protein entries. This large task is made more difficult when the curator cannot easily identify precisely which protein(s) a paper is referring to. This is often due to a lack of information about the species or strain of the source organism of the protein being studied. The simple addition of a UniProt accession number in a paper could go a long way to helping both UniProt and other resources to incorporate the protein knowledge from new papers into their databases. This has many benefits for authors including increasing the reach and impact of their work. We request that authors use the following format (UniProtKB P68369) to describe a protein within the text of a paper. Using this format can also provide a simple mechanism to refer to other data resources. Some journals already have specific formatting requirements for such citations to accessions and these should be given precedence.

### Automatic annotation

**Figure 4.**
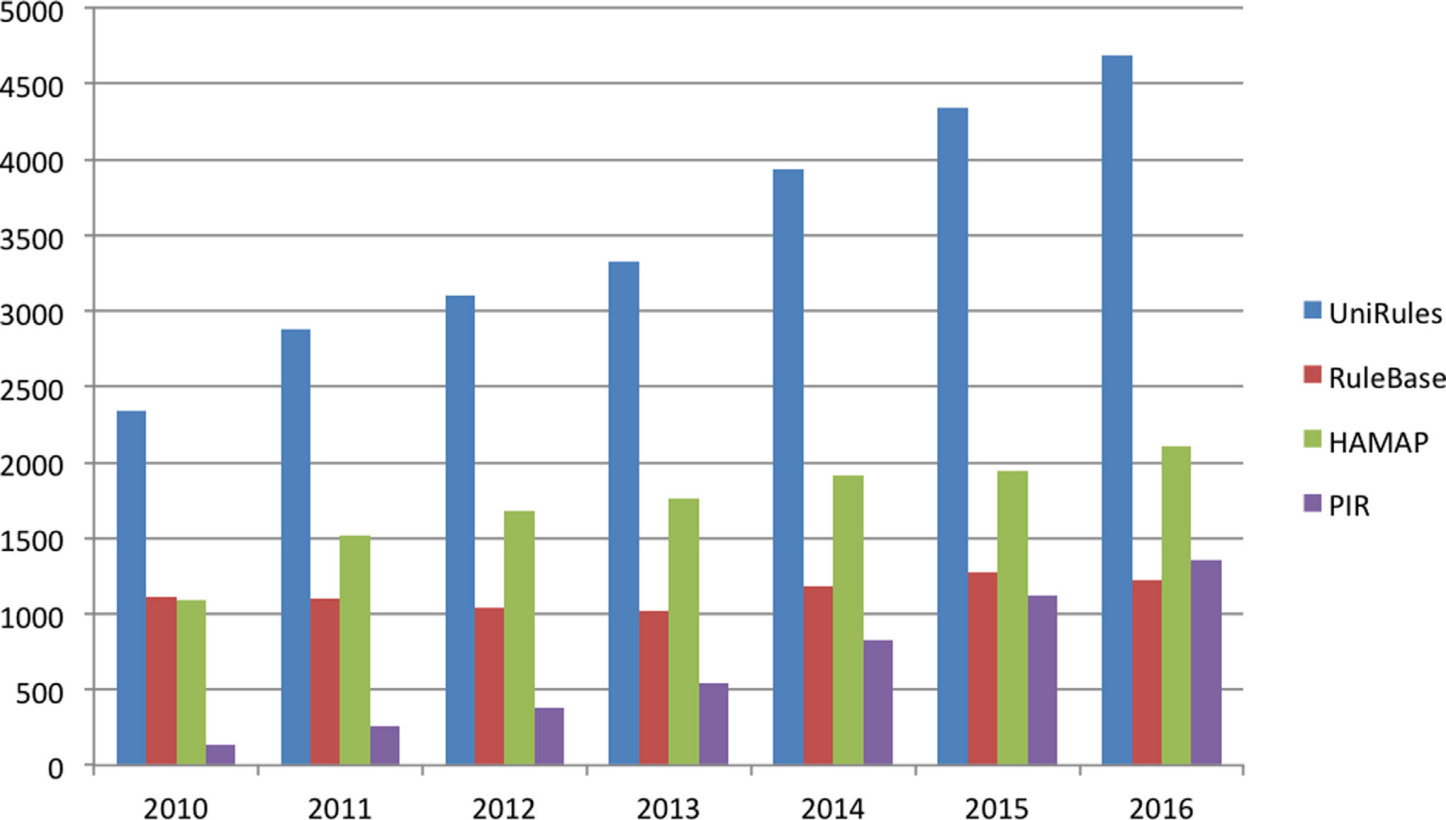
Growth of automatic annotation rules within UniRule. UniRule integrates rules from HAMAP, PIRSF and RuleBase.

We have developed two complementary rule-based systems to automatically annotate the unreviewed protein sequences of UniProtKB/TrEMBL with a high degree of accuracy. The first system, UniRule, consists of annotation rules which are created by the biocurators as part of the process of curation of the experimental literature for UniProtKB/Swiss-Prot. Our priorities for UniRule generation are (i) to focus on using and annotating new functional data of interest for proteomes, such as enzymes and pathways and (ii) to expand our coverage into new taxonomic and protein families. The developers ensure that the supporting technical infrastructure enables these rules to be created, maintained and applied both accurately and efficiently. UniRule is complemented by the Statistical Automatic Annotation System (SAAS), a completely automatic decision-tree-based rule-generating system in which rules are derived automatically from UniProtKB/Swiss-Prot entries sharing common annotations and characteristics. Both UniRule and SAAS use the hierarchical InterPro classification of protein family and domain signatures (18) as a basis for protein classification and functional annotation. These rules share a common syntax that specifies the predicted annotations—including protein nomenclature, function, subcellular location and catalytic residues—and necessary conditions, such as the requirement for conserved functional residues and motifs. InterPro integrates signatures from the HAMAP (19) and PIRSF (20) projects within the UniProt Consortium. Figure 4 illustrates our progress in UniRule generation to date.

In addition to the ongoing development of our rule-based systems, we have also extended our automatic annotation approach in collaboration with the InterPro team at EMBL-EBI by focussing on enriching the feature annotation (FT) of UniProtKB/TrEMBL. The SAMs (Sequence Analysis Methods) are a suite of methods (Coils v2.2, TMHMM v2.0, Phobius and SignalP v4.0 currently) from external providers used to automatically generate sequence features such as Signal, Chain, Transmembrane and Coil regions. The results of these methods are combined and refined according to UniProt standards with the addition of the appropriate UniProtKB annotation. The new predictions are propagated into all the UniProtKB/TrEMBL records that do not already have feature predictions from UniRule. This data was introduced at UniProt release 2015_10 and is reapplied with each UniProt release. Another recent development in this collaboration is the annotation of domain predictions from the InterPro member databases PROSITE, SMART or Pfam in UniProtKB/TrEMBL entries. The name of the domain is taken from UniProtKB/Swiss-Prot annotation or InterPro entry names. This was introduced at UniProt release 2015_12 and is reapplied with each UniProt release. The resulting enrichment of the annotation present in UniProtKB/TrEMBL has increased significantly with these complementary approaches, as has the coverage. Annotation from the automatic annotation systems is labelled with evidence attribution indicating the specific source rule/method on both the UniProt website and in the UniProtKB files available at http://www.uniprot.org/downloads.

### Mapping of proteomic data

High-throughput proteomics experiments deposited in proteomics repositories are a rich source of annotations for UniProtKB, providing supporting evidence for the existence of specific protein isoforms and PTMs. We have developed a pipeline that provides evidence annotations to UniProtKB records using proteomics datasets currently from PeptideAtlas (21) MaxQB (22) and Encyclopedia of Proteome Dynamics (23). Species-specific lists of identified experimental peptides from these repositories are uniquely matched to the products of a single gene by comparison with a database of in-silico digested peptides within the target UniProtKB species. Unique peptide identifications are used to generate annotations in UniProtKB/TrEMBL and to confirm protein existence. All peptide identifications and their mapped UniProtKB proteins are available via the UniProt FTP site (ftp://ftp.uniprot.org/pub/databases/uniprot/current_release/knowledgebase/proteomics_mapping/) and as a dedicated track in the UniProt ProtVista feature viewer in the website. We have used our pipeline to annotate and map experimental peptides to UniProtKB proteins in 10 species including *Homo sapiens, Mus musculus, Rattus norvegicus, S. cerevisiae, S. pombe, Caenorhabditis elegans, Drosophila melanogaster, Danio rerio, Sus scrofa* and *Bos taurus.* Future work will focus on the annotation of PTMs and the extension of mappings to experimental data sets in the PRIDE repository (24) and the Consortium for Top Down Proteomics (25).

### Mapping the UniProt human proteome to the reference genome

**Figure 5.**
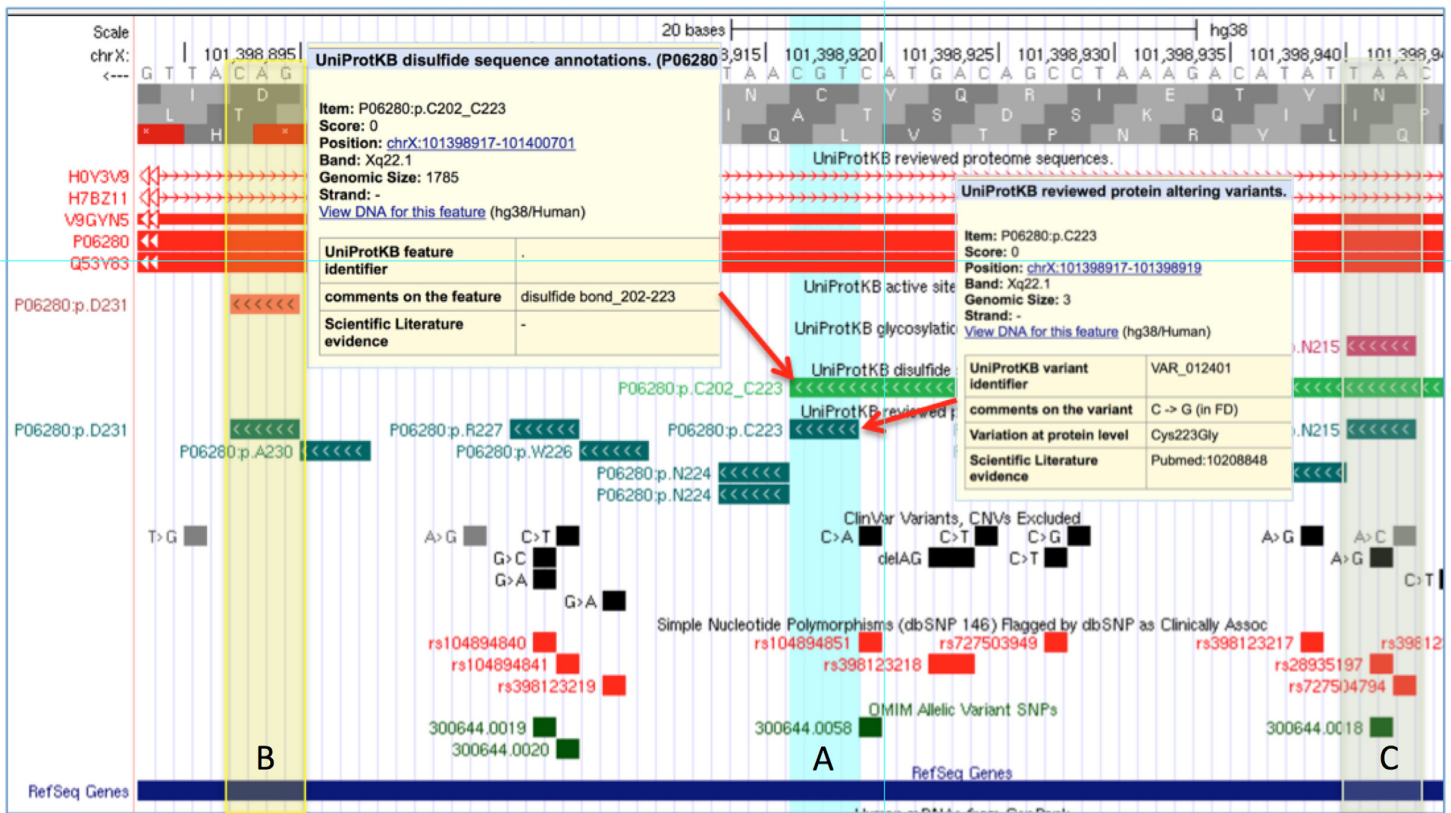
The GLA gene (P06280, α-galactosidase A) associated with Fabry disease (FD) is shown on the UCSC genome browser using UniProt genome tracks plus variations from ClinVar, dbSNP and OMIM. Panel (**A**) shows UniProt annotation for a disulfide bond and an amino acid variation associated with FD that removes the Cystene required for a structural fold. Similar situations exist in panel (**B**) where part of the enzyme's Active Site is disrupted and panel (**C**) where an N-linked carbohydrate is located. Only the pathogenic variation in C is annotated in other public resources.

With the increasing interest and progress in genomic-based medicine understanding the effects of variation on protein function is a key component of gene and variant curation (26,27). Unfortunately much of this information has not been readily available to medical geneticists. UniProtKB contains decades of literature-based and semi-automated curation describing protein function including variation data (28). Standard NGS annotation tools identify simple effects like translation stops and missense mutations, but more subtle effects like disruption of an enzyme active site, protein-binding site or post-translationally modified site are often not included. To make this information available to the genomic community, UniProt in collaboration with Ensembl has now mapped protein sequence annotation in the human reference proteome to the GRCh38 build of the human genome. Figure 5 illustrates pathogenic variants annotated in UniProt but not yet in ClinVar, dbSNP or OMIM. The figure also shows how these variants disrupt protein structural and functional features. Twenty-six positional annotation tracks and a protein sequence track are provided. These include: enzyme active sites; modified residues; protein binding domains; protein isoforms; protein variations; and more. The mappings and related annotation are available on the UniProt FTP site as extended BED text files and binary BigBED files for use on most genome browsers and also programmatically via a REST API (http://www.ebi.ac.uk/proteins/api/doc/swagger/index.html). The mapping and annotation are also available as public Genome tracks on the Ensembl and UCSC genome browsers and via the Track Hub Registry (trackhubregistry.org).

### Website developments

**Figure 6.**
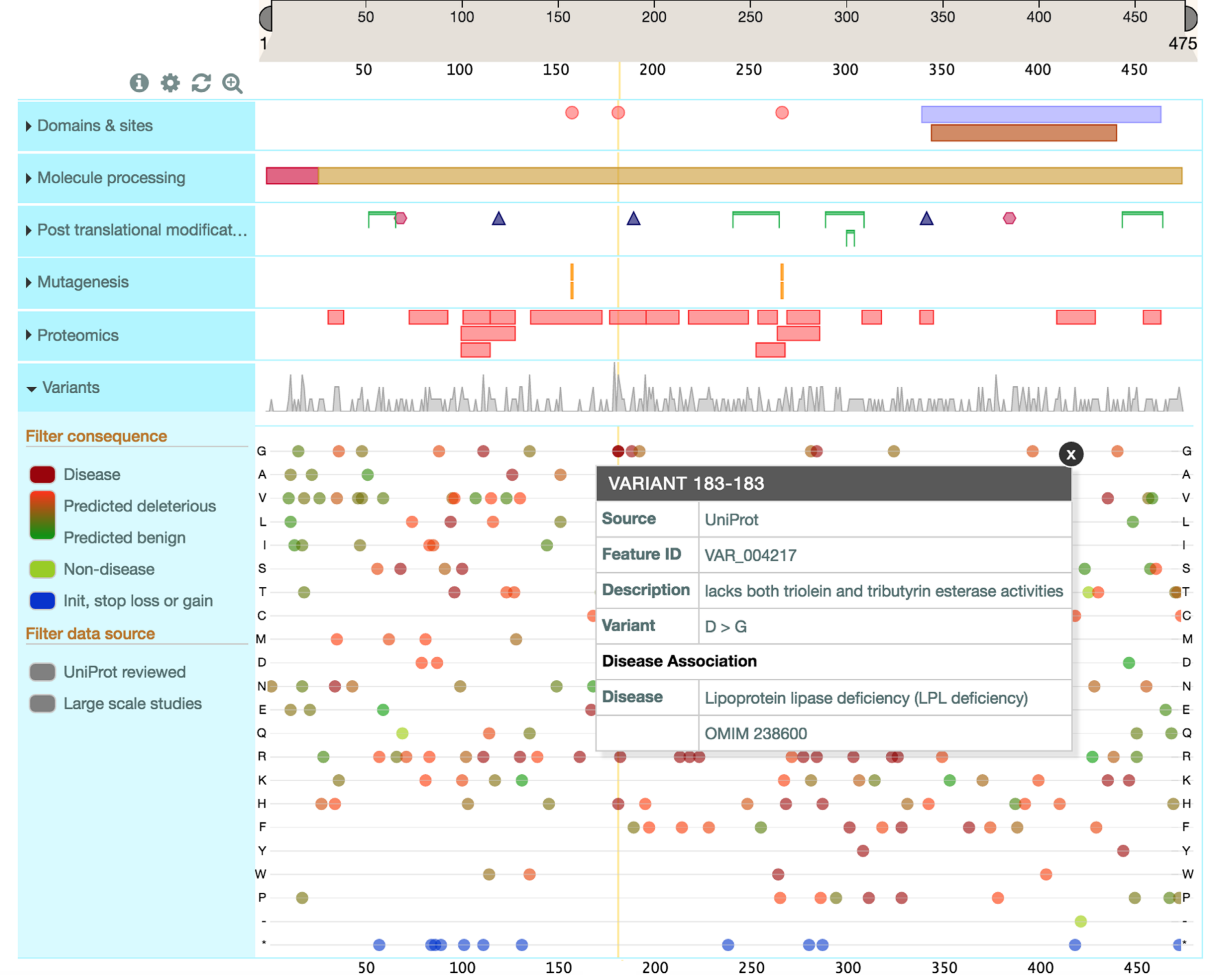
The ProtVista feature viewer. ProtVista uses tracks to display different protein features providing an integrated intuitive picture. The tracks can be expanded, as shown in this Figure with the Variants track. Clicking on a feature highlights its position across all tracks so that co-localized elements can be easily identified. For example here the highlighted site is at the same position as disease correlated natural variants.

UniProt follows a user-centered design process, involving many users worldwide with varied research backgrounds and use cases, to improve its website and add new features. The most notable new development is the ProtVista feature viewer, a BioJS component that brings together sequence annotations in one compact view. UniProt FTs describe sites and regions of biological interest, such as an enzyme's active site, binding sites, PTMs, domains, etc., which play a critical role in the understanding of what the protein does. With the growth in biological data, integration and visualization become increasingly important for exposing different data aspects. Similar to genome browsers, ProtVista uses tracks to display different protein features, providing an intuitive picture of co-localized elements (see Figure 6).

Each track can be expanded to reveal a more in-depth view of the underlying data. Users can zoom into and out of an area and clicking on a feature will trigger a pop-up with more information about the feature, such as the feature position, description and any available evidence. The view can be customized to hide or show feature tracks. The viewer shows all features of a UniProtKB entry and includes additional features that are mapped from large-scale studies, currently for variants and proteomics data. The viewer is available for every UniProtKB protein entry through the ‘Feature viewer’ link under the ‘Display’ heading on the left hand side of the entry page.

The UniProt proteome pages (http://www.uniprot.org/proteomes) were launched in 2015 to enable users to access proteomes for species with completely sequenced genomes. One can query the data by taxonomy as well as genome and proteome identifiers and filter results for reference or non-redundant proteomes. The results table indicates the number of UniProt entries for each proteome and allows users to view or download them in a range of formats.

**Figure 7.**
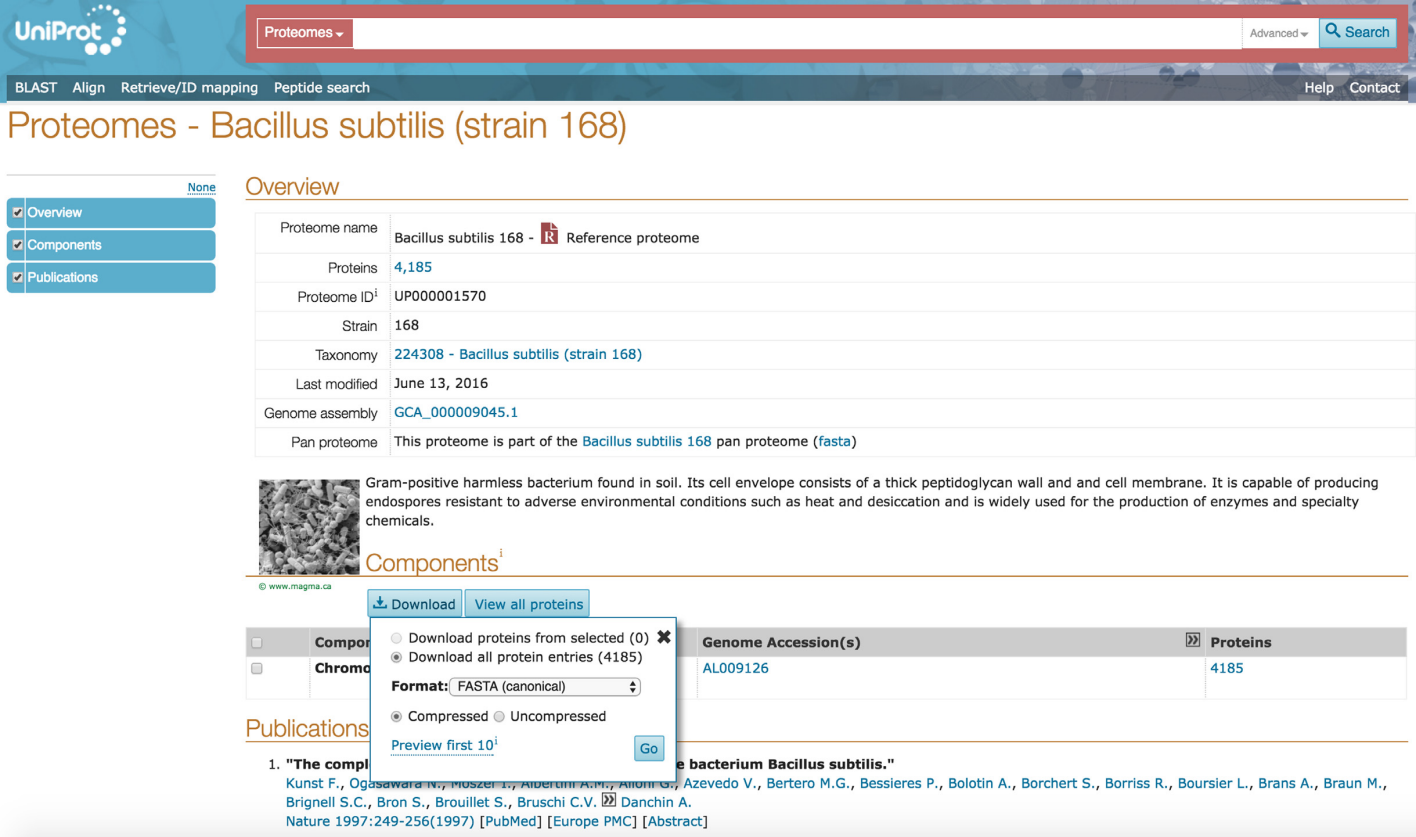
Proteome page for *Bacillus subtilis* 168. Proteome pages contain a short overview with details about the organism and genome assembly, the list of the genome's components and references from the sequencing projects.

Individual proteome pages provide a short overview with details about the organism and genome assembly. The components of the genome assembly are listed in a table where it is possible to view or download the UniProt entries for selected components or the entire proteome. If a proteome is part of a pan proteome, a download link for the pan proteome is also provided. The pages for redundant proteomes link to the corresponding non-redundant proteome. Relevant publications describing the sequencing of the genome are also listed. Figure 7 shows the proteome page for *B. subtilis 168*—a non-redundant proteome. A short video tutorial is available on the UniProt YouTube channel (https://www.youtube.com/watch?v=ZLt3ug0mZ7A).

**Figure 8.**
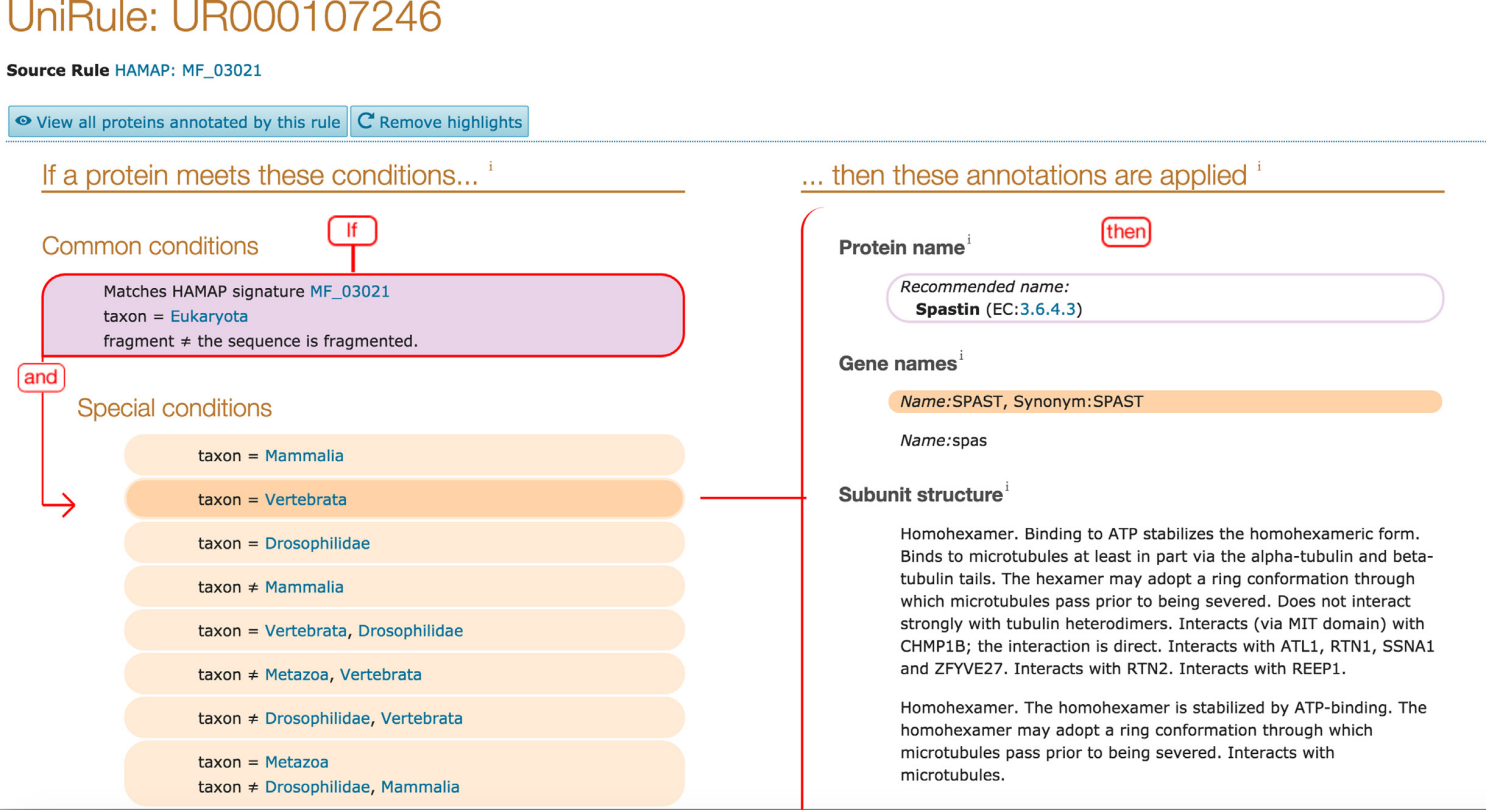
An example UniRule entry page. Clicking on the conditions highlights the corresponding annotations applied if the conditions hold true and *vice versa* clicking on the annotations highlights the corresponding conditions. Clicking on the ‘View all proteins annotated by this rule’ button leads to the list of proteins that this rule annotates in UniProtKB.

We have also added new pages for UniProt's automatic annotation systems, UniRule (Unified Rule system) and SAAS, which are used to automatically annotate unreviewed UniProtKB/TrEMBL entries in an efficient and scalable manner. All annotations that are predicted in this way link back to their source rules and users can now inspect these rules to understand which conditions have generated the annotations and to find other proteins that have been annotated by the same rule (see Figure 8).

**Figure 9.**
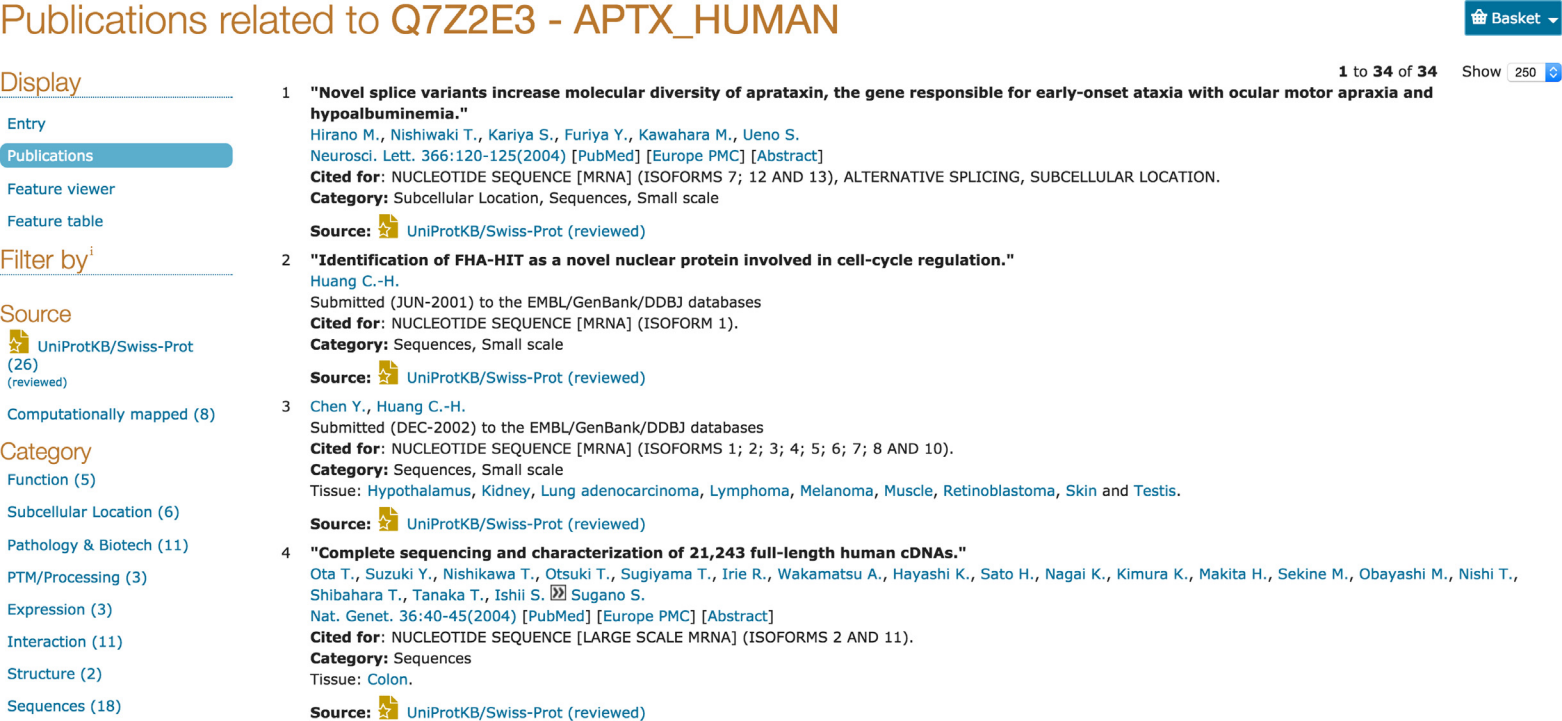
The new publications view for UniProtKB entries.

UniProtKB protein entries now have an enhanced view of publications relevant for a protein. UniProtKB contains more than 350 000 unique publications, with over 210 000 of these fully curated in UniProtKB/Swiss-Prot and the remainder imported in UniProtKB/TrEMBL. This set is complemented by more than 640 000 additional publications that have been computationally mapped from other resources to UniProtKB entries. The publications annotated in UniProtKB have previously been displayed in the entry view and a link provided access to a separate page that listed the computationally mapped publications. We have now combined all publications into a new ‘Publications’ view that can be accessed from a link under the ‘Display’ section (see Figure 9). This has made it possible to implement filters to narrow the publication list by categories that are based on the type of data a publication contains about the protein (such as function, interaction, sequence, etc.) and it is easy to see if there are mapped publications in a category for which no annotated publications are available.

**Figure 10.**
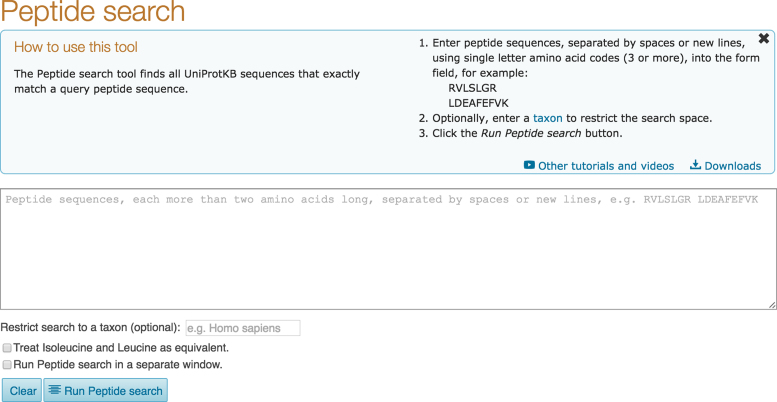
The Peptide search interface.

We have also introduced a new tool called ‘Peptide search’ (see Figure 10) that is available from a link in the header of the UniProt website. Users can enter a peptide sequence (for example from a proteomics experiment) into the search field and the tool quickly finds all UniProtKB sequences that match 100% with the query sequence (29). Searches can be restricted to a taxonomic subset of UniProtKB to further decrease the search time. The tool returns a results page showing the matched UniProtKB entries in a design consistent with the UniProtKB text search results page, including filters on the left, results on the right and an option to customize the results table through the ‘Columns’ button.

### SPARQL endpoint

UniProt has been distributing its databases in RDF format (*https://www.w3.org/RDF/*) since 2007. Complete datasets can be download from the UniProt FTP sites and individual records and query result sets can be retrieved programmatically via the UniProt website's REST API. To allow our users to also query these RDF data with SPARQL (https://www.w3.org/TR/rdf-sparql-query/), we have collaborated with several database software vendors and provided test datasets and use cases for performance studies to help them improve their RDF storage products over the years. The main challenges for the application of this technology are the data volume (22 billion triples at UniProt release 2016_07) and the highly variable complexity and number of user queries (with monthly averages ranging from app. 90 000 to over 4 million in 2015). By 2014, several RDF storage solutions had reached a stage of maturity that made it feasible to implement a public SPARQL endpoint for beta testing. This allowed us to test the technical infrastructure and also to identify areas for improvements in the data representation. The http://sparql.uniprot.org/ server has been running in production since July 2015 and is used by over 1000 unique users every month. It is updated with every UniProt release and can be linked using SPARQL 1.1’s federated query capabilities with any remote data resource that has a SPARQL endpoint.

## CONCLUSIONS

Our knowledge of the protein world is rapidly growing, and the complexity of biological systems is becoming increasingly clear. UniProt continues to adapt its data gathering, data processing and data display to improve the availability and utility of protein information for the benefit of all.

The user community can contact UniProt with feedback and queries through the ‘Contact’ link on the website or via the e-mail help@uniprot.org. They can also subscribe to our Twitter feed @UniProt, follow our Facebook page or follow our blog ‘Inside UniProt’ for the latest updates.
