# UniProt: the universal protein knowledgebase

**DOI:** 10.1093/nar/gky092

**Published:** 2018-02-07

**Authors:** The UniProt Consortium

**Affiliations:** 1 European Molecular Biology Laboratory, European Bioinformatics Institute (EMBL-EBI), Wellcome Genome Campus, Hinxton, Cambridge CB10 1SD, UK; 2 SIB Swiss Institute of Bioinformatics, Centre Medical Universitaire, 1 rue Michel Servet, 1211 Geneva 4, Switzerland; 3 Protein Information Resource, Georgetown University Medical Center, 3300 Whitehaven Street NW, Suite 1200, WA 20007, USA; 4 Protein Information Resource, University of Delaware, 15 Innovation Way, Suite 205, Newark DE 19711, USA

*Nucleic Acids Research* (2017) 45: D158–D169, https://doi.org/10.1093/nar/gkw1099

The authors wish to make the following correction to their article. Co-author Alexandre Renaux affiliated with the EMBL-EBI was mistakenly omitted from the list of authors. His name has been added to the full list of authors in the Acknowledgement section of the published article.

